# Arsenic and the Placental Epigenome: Unlocking the Secrets of Prenatal Exposure

**DOI:** 10.1289/ehp.124-A148

**Published:** 2016-08-01

**Authors:** Lindsey Konkel

**Affiliations:** Lindsey Konkel is a New Jersey–based journalist who reports on science, health, and the environment.

Experts suspect that epigenetic changes such as DNA methylation may be involved in adverse health effects associated with fetal arsenic exposure.^1^ Previous studies have investigated associations between arsenic exposure and DNA methylation in adult and umbilical cord blood cells.^1^ ^,^ ^2^ ^,^ ^3^ Now researchers present an extensive epigenome-wide analysis of placental DNA methylation in relation to fetal arsenic exposure.^4^

Methylation varies among different cells and tissues, so past DNA methylation studies have been limited by their reliance on blood cells. By studying methylation in placental cells, investigators are able to measure variations in a tissue where it may have specific impacts on health outcomes.

**Figure:**
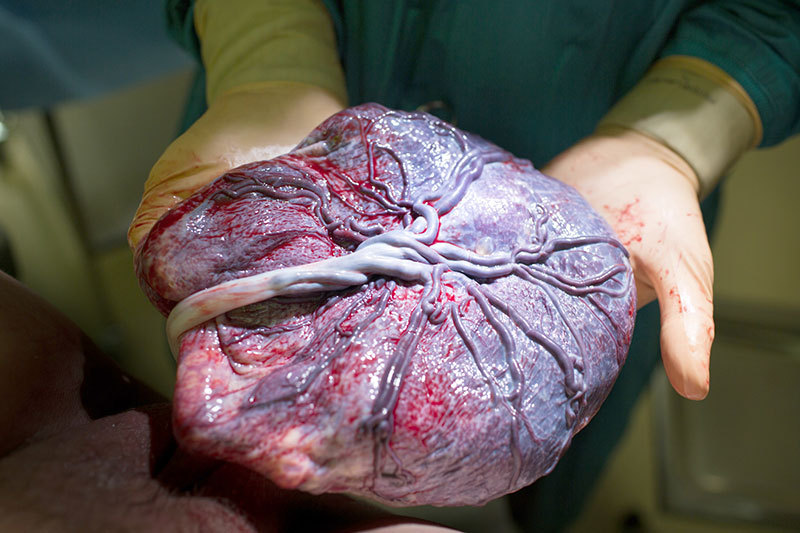
The placenta is a rich source of information on fetal exposures and development. © Kurt Drubbel/Getty Images

The placenta, which connects the developing fetus to the uterine wall, largely controls the fetal environment.^5^ It transfers nutrients to the fetus and shuttles waste products out. It regulates fetal interactions with the maternal immune system and exposure to compounds in the mother’s blood. The placenta also acts as a neuroendocrine organ, producing hormones, growth factors, and cytokines.^6^

“If the expression of genes that are involved in these important processes become altered in some way, fetal development and long-term health outcomes may be impacted,” says senior author Carmen Marsit, an environmental epigeneticist now at Emory University. Marsit conducted the work while at Dartmouth’s Geisel School of Medicine.

The current study, led by postdoctoral fellow Benjamin Green, compared arsenic exposure with placental genome-wide DNA methylation profiles for 343 mother–infant pairs enrolled in the New Hampshire Birth Cohort Study. Arsenic was measured in the mothers’ urine and toenail clippings and in the expelled placenta. Naturally occurring arsenic contamination of drinking well water was believed to be a major source of exposure for mothers, although food-based exposures likely also contributed.^4^

Arsenic measured in maternal urine and toenail clippings did not predict methylation in placental cells. However, higher arsenic levels in placental samples were associated with altered methylation levels at 163 different DNA regions. In particular, the researchers found 11 methylation markers in the *LYRM2* gene, and methylation decreased at all 11 markers as arsenic exposure increased. When they measured *LYRM2* mRNA levels they found that decreased DNA methylation at the targeted loci was associated with expression of the gene.^4^

It’s not yet known what function *LYRM2* might perform in the placenta, although other genes in the same family play an important role in the synthesis of proteins that help to metabolize metals.^7^ Researchers are now working to understand the functional implications of the observed epigenetic differences and what they might mean for fetal growth.

False positives are a concern in high-throughput DNA analyses that make hundreds of thousands of comparisons, says Robert Wright, a pediatrician and environmental epigeneticist at the Icahn School of Medicine at Mount Sinai, so it’s reassuring to see multiple hits in one gene. “It increases confidence that these results are real,” says Wright, who was not involved in the study.

Environmental chemicals that are deposited in the placenta during pregnancy often stay there, says Wright. The implication is that levels of chemicals in the delivered placenta represent cumulative exposures over the course of the whole pregnancy. By comparison, urine reflects relatively recent exposures, while toenail clippings reflect exposures that occurred months before (i.e., the amount of time it took the nail to reach a clippable length). “The potential of the placenta to quantify exposure in pregnancy has been underestimated, and this study demonstrates its value,” Wright says.

Placenta studies like this one represent the forefront in environmental epigenetics, according to Wright. Like blood or urine, the placenta is a very accessible tissue—most are simply discarded after birth. But the placenta also grows and expresses growth factors. This may make it a better surrogate for target tissues that also grow during development, such as the heart, brain, or liver, Wright says.

That said, epigenetic differences among placentas don’t necessarily reflect differences in other tissues, although they may elucidate the effects of environmental factors on various molecular pathways. Says Marsit, “The placenta may offer a unique way to study how environmental exposures alter the growth and development of harder-to-reach tissues on the molecular level.”
